# Supplementary material for: Mild Chemical Treatment of Unsorted Urban Food Wastes
Source: Molecules. 2023 Nov 20;28(22):7670. doi: 10.3390/molecules28227670 (PMC10674513; doi:10.3390/molecules28227670)
Supplement: Supplementary file 1 [file molecules-28-07670-s001.zip › molecules-2689130-supplementary.pdf]

Table S1. Data (% w/w referred to dry matter) for mass, C and N balance for fractions (Figure 2) isolated by membrane ultrafiltration of crude soluble matter listed in Table 2 obtained by centrifugation, hydrolysis and ozonisation of as collected MBW: i.e. the retentates at 750 kDa (R750), 100 kDa (R100), 20 kDa (R20), 0.2 kDa (R0.2) and the final permeate at 0.2 kDa (P0.2).

**Mass yield**	**Centrifugation**	**Hydrolysis**	**Ozonisation**
R750	3.6	27.1	13.1
R100		0.9	1.1
R20	0.3	0.9	0.8
R5	5.5	5.7	
R0.2			3.0
P0.2	90.6	65.4	82.0
C yield			
R750	5.6	40.6	26.6
R100		1.2	1.8
R20		1.0	1.8
R5	5.7	7.9	
R0.2			4.9
P0.2	88.7	49.3	64.9
N yield			
R750	8.1	30.9	86.9
R100		1.0	5.6
R20		1.5	2.6
R5	5.8	18.8	
R0.2			1.3
P0.2	86.1	47.8	4.3
Fractions C content			
R750	46.1	41.4	39.0
R100		38.1	33.8
R20		34.0	42.2
R5	30.7	38.6	
R0.2			30.17
P0.2	29.0	22.2	15.1
Fractions N content			
R750	5.33	1.80	6.58
R100		1.69	4.47
R20		2.82	3.20
R5	2.52	5.20	
R0.2			4.17
P0.2	2.24	1.15	0.48
C/N			
R750	8.6	22.9	5.9
R100		22.6	7.4
R20		12.1	13.2
R5	12.2	7.4	
R0.2			7.2
P0.2	12.9	19.3	31.4

Table S2. C types and functional groups[a] relative composition[b] as % mole/mole relative to total organic C for Table 1 MBW proximates, for crude soluble and insoluble products in Table 2 and for the retentates and permeates fractions of the crude soluble products in Figure 2 and 3.

**Signal chemical shift (δ, ppm)**	0-53	53-63	63-95	95-110	110-140	140-160	160-185
**Product/C type[a]**	Af	NR	OR	OCO	Ph	PhOY	COX
Lipids (Table 1)	92.7	0.82	0	0	2.74	0	3.70
Mono-/disaccharides (Table 1)	28.0	8.82	38.7	4.14	n.d.[c]	n.d.[c]	12.1
Hemicellulose/protein (Table 1)	34.9	6.39	16.1	4.28	13.4	n.d.[c]	22.4
Lignin (Table 1)	13.6	9.44	35.6	8.48	12.7	9.13	5.51
Pristine MBWS SM (Table 2)	32.6	6.05	28.9	2.92	8.24	n.d.[c]	17.1
Pristine MBWS IM (Table 2)	19.4	7.94	48.1	9.77	4.96	n.d.[c]	7.46
Hydrolysed SM (Table 2)	52.0	n.d.[c]	12.1	1.33	10.2	n.d.[c]	16.2
Hydrolysed IM (Table 2)	39.6	3.45	32.7	7.17	6.40	n.d.[c]	9.86
Ozonized SM (Table 2)	37.2	n.d.[c]	9.56	3.00	n.d.[c]	n.d.[c]	22.7
Ozonized IM (Table 2)	23.9	8.02	46.3	9.21	n.d.[c]	n.d.[c]	7.91
Pristine MBWS R750 (Table 3)	48.5	12.9	17.1	5.06	4.07	n.d.[c]	11.0
Hydrolysed R750 (Table 3)	43.1	6.08	30.1	6.18	6.55	n.d.[c]	7.22
Ozonised R750 (Table 3)	69.4	n.d.[c]	7.54	n.d.[c]	n.d.[c]	n.d.[c]	15.2
Ozonised R100 0 (Table 3)	46.4	8.29	20.1	4.10	4.02	n.d.[c]	16.6

[a]Data for aliphatic and/or cycloaliphatic (Af), amine (NR), alkoxy (OR), anomeric (OCO), aromatic (Ph), phenol and phenoxy (PhOY, Y = H and/or R), carboxylic and amide (COX, X = OR, OM, NR-) C, R = H, alkyl and/or aryl C. [b]see Materials and Methods section. [c]not distiguished fom the background noise.

Table S3. Relative chemical composition of water soluble products (see Figure 4) obtained with the multistep treatment in Figure 1B [12, 21].

**Signal chemical shift (δ, ppm)**	0-53	53-63	63-95	95-110	110-160	160-185	MW kDa
**Product[a]/C type[b] and MW**	Af	NR	OR	OCO	Ph/PhOY	COX	
Hydrolysis crude soluble product from anaerobic digestate [21]	43	10	14	3	13	16	
Hydrolysis soluble R_{750} from anaerobic digestate [21]	49	8	13	4	8	14	≥ 750
Hydrolysis soluble R_{150} from anaerobic digestate [21]	42	10	15	4	11	18	150-750
Hydrolysis soluble R_{100} from anaerobic digestate [21]	39	10	11	3	12	21	100-150
Ozonisation soluble R_{750} from anaerobic digestate [21]	74	1	3	3	9	6	≥ 750
Ozonisation soluble R_{150} from anaerobic digestate $_s$ [21]	73	3	1	2	8	8	150-750
Ozonisation soluble R_{100} from anaerobic digestate [21]	50	6	8		7	26	100-150
Hydrolysis crude soluble product from from CGD compost [12]	41	7	14	4	18	15	77
Hydrolysis soluble R_{35} from CGD compost [12]	22	9	9	3	33	23	279
Ozonisation crude soluble product from CGD compost [12]	20	6	3	2	20	48	
Ozonisation soluble R35 from CGD compost [12]	62	5	9	5	5	13	487
Hydrolysis crude soluble product from CG compost [12]	37	7	14	4	20	18	80

Hydrolysis soluble R35 from GG compost [12]	27	8	8	3	32	23	18
Ozonisation crude soluble GG compost [12]	17	6	7	3	22	44	
Ozonisation soluble R35 from CG compost [11]	50	10	13	3	5	18	223

[a] Legends as for Figure 4.

Table S4. Surface tension (γ, mN m^{-1})[a] of products in Figure 3 and 4.

**Product**	γ, mN m^{-1}
Centrifugation soluble R750 (Figure 3C)	47.1±0.2 a
Hydrolysis soluble R750 (Figure 3C)	42.2±0.2 b
Ozonisation soluble R750 (Figure 3C	54.0±0.1 c
Hydrolysis soluble R35 from CGD compost (Figure 4A)	59.8±0.1 d
Hydrolysis soluble R35 from CG compost (Figure 4A)	59.2±0.2 e
Hydrolysis soluble R750 from anaerobic digestate (Figure 4A)	56.8±0.3 f
Ozonisation soluble R750 from anaerobic digestate (Figure 4B)	39.5±1.0 g
Hydrolysis soluble R150 from anaerobic digestate (Figure 4A)	57.3±0.2 f
Ozonisation soluble R150 from anaerobic digestate (Figure4B	36.8±0.6 g
Hydrolysis soluble R100 from anaerobic digestate (Figure 4A)	54.2±0.3 h
Ozonisation soluble R100 from anaerobic digestat (Figure 4B)	43.6±2.3 i

[a]Values followed by different letters are significantly different at P<0.01 level
